# Shape Memory Epoxy Resin and Its Composites: From Materials to Applications

**DOI:** 10.34133/2022/9767830

**Published:** 2022-03-16

**Authors:** Lan Luo, Fenghua Zhang, Jinsong Leng

**Affiliations:** Centre for Composite Materials and Structures, Harbin Institute of Technology (HIT), Harbin 150080, China

## Abstract

Shape memory polymers (SMPs) have historically attracted attention for their unique stimulation-responsive and variable stiffness and have made notable progress in aerospace, civil industry, and other fields. In particular, epoxy resin (EP) has great potential due to its excellent mechanical properties, fatigue resistance, and radiation resistance. Herein, we focus on the molecular design and network construction of shape memory epoxy resins (SMEPs) to provide opportunities for performance and functional regulation. Multifunctional and high-performance SMEPs are introduced in detail, including multiple SMEPs, two-way SMEPs, outstanding toughness, and temperature resistance. Finally, emerging applications of SMEPs and their composites in aerospace, four-dimensional printing, and self-healing are demonstrated. Based on this, we point out the challenges ahead and how SMEPs can integrate performance and versatility to meet the needs of technological development.

## 1. Introduction

Shape memory polymers (SMPs) are stimuli-responsive novel smart materials. They have both perceptual and driving functions under ambient conditions and temporary and original shapes in the process of deformation under external stimuli [1–3]. SMPs are fixed in a temporary shape under external and environmental conditions and restored to their original shape (permanent shape) under specific stimuli. SMPs can be divided into different types according to different stimulus responses such as heat [4, 5], light [6–8], humidity [9], electric field [10, 11], and magnetic field [12, 13]. Although many stimuli trigger shape memory effects (SME), most are produced by direct or indirect heating. According to the number of temporary shapes in the shape memory cycle, SMPs can be divided into dual-SMP [14, 15], triple-SMP [16, 17], and multiple-SMP [18, 19]. Multi-SMEPs exhibit one-way or irreversible shape memory effect, which is one-way SMPs (1W-SMPs). In contrast, two-way SMPs (2W-SMPs) exhibit reversible shape switching between different shapes, which is programmable [17, 20–22]. SMP has the characteristics of large deformation, variable stiffness, and large shrinkage, ranging from oil exploitation and sealing to drug delivery. Therefore, in aerospace, its high compression ratio saves storage space [23–25] and provides the driving function for artificial muscle in intelligent bionics. In addition, it has good biocompatibility and is widely used in biomedical and other fields [26–28]. In recent years, it has been favored by more and more researchers.

Epoxy resin (EP) has excellent mechanical properties and corrosion resistance, and shape memory epoxy resins (SMEPs) are used in most fields. The shape memory phenomenon in SMEPs satisfies the following two structural features: (1) crosslinks to determine the permanent shape and (2) reversible phases with a transition temperature (*T*_trans_) to fix the temporary shape [29]. The most common SMP is a thermally stimulated one-way SMP. At low temperature, the molecular chains are frozen and polymers are stiff. However, when heated above *T*_trans_, they become soft rubbery (or elastomer) due to the increased movement of molecular chains. The polymer network structures are thought to be constructed through crosslinking points, maintaining a stable shape on the macroscopic level. The domains of the crosslinking points can be physically or chemically crosslinked [20]. Physically crosslinked polymers (thermoplastics) have reversible properties which melt or dissolve in certain solutions. The formation achieves the interconnection between individual polymer chains forming a crystalline or glass phase. The individual polymer chains are linked by covalent bonds for chemically crosslinked polymers. They are more stable than physically crosslinked networks and have an irreversible nature.

**Figure 1:**
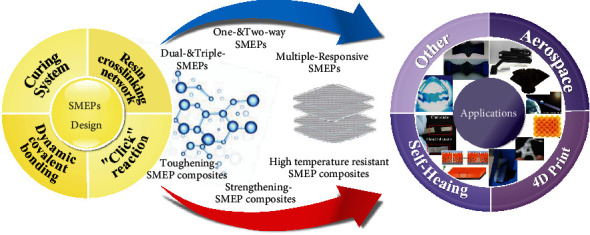
Multiple functional shape memory epoxy composites: from materials to applications.

In recent years, SMEPs have continuously penetrated into the aerospace, industrial, and civilian fields and entered other new industries. In this paper, we review a class of smart materials known as SMEPs which exhibit shape change and shape recovery properties in response to various external stimuli. The latest development of a micromolecular design is summarized, and the relationship between micromolecular design and macroscopic performance, multifunction, and application is emphasized. Multifunctional SMEPs with multiple responsive, multishape, and two-way shape memory effects move towards new materials to meet multiple needs. High-performance SMEPs with high strength, toughness, and heat resistance have opened up new heights for various applications, as shown in Figure 1. SMEPs offer tremendous prospects for smart products in many fields of science and technology in the future.

## 2. SMEP Formulations

**Figure 2:**
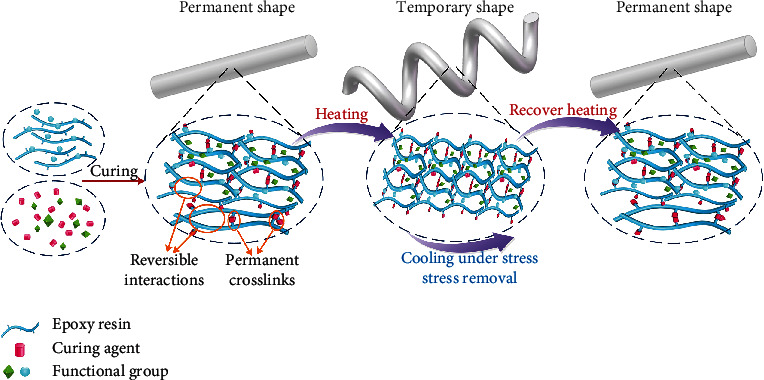
Schematic diagram of shape memory mechanism of epoxy resin.

Epoxy resin is a thermosetting polymer that forms a three-dimensional network when an epoxy resin containing at least two epoxy groups reacts with a curing agent. According to different formulation systems and curing conditions, various curing resins with controllable characteristics can be obtained. Combining different resins and curing agents (at various resins, curing agent ratios, and curing methods) with additives (such as diluents, fillers, or tougheners) has developed in the direction of refinement and serial functionalization. The mechanism of SMEP synthesis is shown in Figure 2.

### 2.1. SMEP Crosslinking Network

The crosslinking network of SMEP determines the properties of the resin itself, such as glass transition temperature (*T*_g_), strength, and elongation at break. The crosslinking network can also be doped with other resins to improve these properties via functional groups or epoxy functionality of the resin itself.

#### 2.1.1. Internal Synthesis

**Figure 3:**
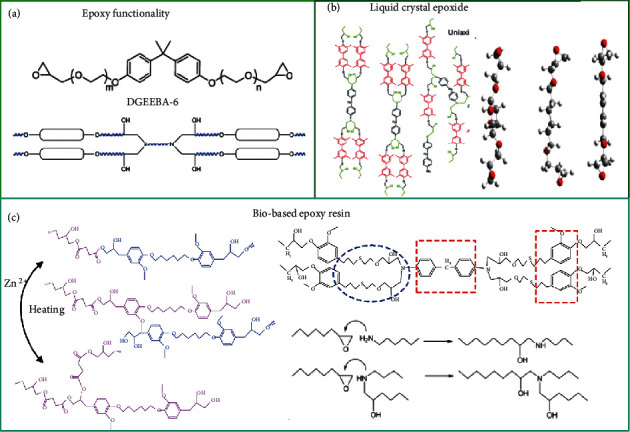
Internal modification of epoxy resin. (a) Multiple functional epoxy groups. Reproduced from Ref. [31]. (b) Liquid crystal epoxy resin. Reproduced from Ref. [32, 34]. (c) Biobased epoxy resin. Reproduced from Ref. [33, 35].

The functionality of the resin can determine the degree of crosslinking. The resin internal crosslinking network design can directly affect the macromechanical properties. Through the design, the toughness, strength and heat resistance of the resin can be adjusted, and more comprehensive properties and functions can be given. Fan et al. synthesized bisphenol A diglycidyl ether (DGEBA) containing two propylene oxide units (DGEBAPO-2) [30]. Combined with the flexible curing agent to obtain an inherent toughening network, the elongation at break and the tensile stress reached 95.53% and 6.33 MPa, respectively. Jo et al. prepared a DGEBA containing six ethylene oxide units to improve *T*_g_ and mechanical properties for better application in space structures [31]. With the increase in DGEBA-6 content, the crosslinking density increases and *T*_g_ decreases, as shown in Figure 3(a). Likewise, liquid crystal epoxide (LCE) is a directed crosslinking network that can improve *T*_g_ and water resistance by introducing biphenyl mesocrystalline materials into the epoxy system. Guo et al. prepared hydrophobic shape memory materials by introducing mesogenic units [32]. The stress orientation of biphenyl leads to an increase in the density of the crosslinked network. Nonplanar ring structures improve the impact strength of thermosetting plastics due to their conformational transformation, and their inherent stiffness can increase the *T*_g_ of EP. Li et al. used nonplanar ring structures of epoxy resins and curing agents to achieve a shape memory polymer with ultrafast shape recovery speed and excellent thermal properties, as shown in Figure 3(b) [33].

Biobased epoxy resins have made a significant progress in recent years. In particular, eugenol-derived epoxy monomers can yield many comprehensive properties and functions, as shown in Figure 3(c). Liu et al. prepared eugenol-derived EP and succinic anhydride in a certain proportion to obtain the crosslinked anhydride curing network that had reprocessability and healing ability [34]. Tian et al. [33] mixed diglycidyl ether of eugenol-2-mercaptoethanol (DGEEM) and diglycidyl ether of vanillic alcohol (DGEVA) to form a rigid-flexible epoxy resin and found that the bicontinuous phase structure and sea-island structure had good mechanical properties. David et al. synthesized a series of shape memory biobased epoxy resins with higher *T*_g_ values by using safe and environmentally friendly eugenol triglycidylphloroglucinol (3EPOPh) and trimethylolpropane triglycidyl ether (TPTE) as epoxy monomers instead of DGEBA [35].

#### 2.1.2. External Doped

When mixed with different functional polymers, such as cyanate ester, polyurethane (PU), and benzoxazine resin, EP is transformed to a more comprehensively functional form exhibiting triple-SME, photothermal conversion, and electrical conductivity properties.

**Figure 4:**
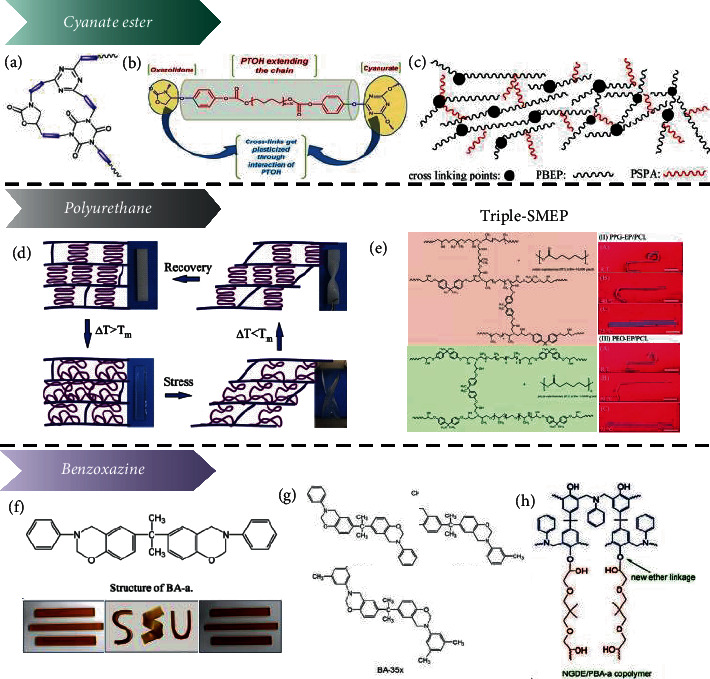
(a–c) Epoxy resin doped with other resins: doped cyanate. Reproduced from Ref. [36, 38, 40]. (d, e) Doped polyurethane. Reproduced from Ref. [42, 43]. (f–h) Doped benzoxazine. Reproduced from Ref. [46–48].

Due to the high *T*_g_ of cyanate ester and its good heat resistance, by copolymerizing it with EP, it is expected to obtain a SMP that meets the requirements of aerospace and other special fields, including high-temperature resistance and excellent overall performance. Kumar et al. synthesized a series of polyether oligomers from epoxy resin and cyanate ester monomer, as shown in Figure 4(a) [36]. An epoxy resin/cyanate crosslinked network was synthesized from polyethylene glycol (PEG), polypropylene glycol (PPG), and polybutadiene glycol (PIG) with *T*_g_ values of 132°C, 178°C, and 161°C, respectively. Biju et al. synthesized SMEPs from bisphenol A dicyanate (BADC), DGEBA, and phenolic distal chelate tetramethoxy compound (PTOH) [37]. And as the cyanate content increases, the system's *T*_trans_ increases, which can be used to develop intelligent actuators [38], as shown in Figure 4(b). Then, they investigated the effect of phenol-terminated oligomers on epoxy-cyanate systems. The higher the oligomer concentration, the lower the transition temperature of the system. Wang et al. [39] prepared SMPs from epoxy resin-polybutadiene epoxy resin (PBEP) and bisphenol A cyanate ester (BACE). The addition of PBEP enabled the fastest recovery rate to 0.0128 s^−1^ and filled with carbon black (CB) for the synthesis of electroactive shape memory polymer composites (SMPCs) [40]. Subsequently, a new crosslink network was formed based on BACE/PBEP with polysebacic acid anhydride (PSPA) [41], and the shape recovery time decreased with the increase of PSPA content, as shown in Figure 4(c).

Polycaprolactone (PCL) is one of the ideal hybrid materials for EP. Triple-SMEP systems generally have a wide *T*_g_ range or generate two independent *T*_g_ peak regions. Torbati et al. [42] prepared EP/PCL mixtures as semicrystalline elastomer and highly rigid amorphous EP by polymerization-induced phase separation (PIPS), both of which showed three temperature plateaus required for the TSME, as shown in Figure 4(e). The concentration of PCL in the crosslinked polymer affected crystalline interactions. Luetzen et al. [43] added EP to random copolymer poly(ethylene glycol-propylene glycol) (PEG-ran-PPG or RCP) [44]. The *T*_g_ value of the system can be adjusted from 61°C to 141°C by changing the concentration of the random copolymer, as shown in Figure 4(d). Besides, Puig et al. [45] dispersed the PE-b-PEO block copolymer in DGEBA for tertiary amine curing. During the cooling process, the nanostructures of PE block crystals self-assembled in the rubber-like region of the epoxy network.

A benzoxazine resin is a thermosetting resin with good comprehensive properties. It has a high *T*_g_, high thermal stability, and excellent processing ability. At the same time, the resin can also be used as a curing agent for epoxy and has a synergistic effect with epoxy. Rimdusit et al. mixed BA-a benzoxazine monomer, epoxy resins, and amine curing agent to produce a new SMP system [46–48]. They had higher bending strength and bending modulus, as shown in Figure 4(f). Subsequently, they used aniline-based benzoxazine resin (BA-a) to increase the stiffness of the crosslinked network, as shown in Figure 4(h). The *T*_g_ value of the system increased with the increase of BA-a content [49].

### 2.2. SMEP Curing System

#### 2.2.1. Amine Curing SMEP System

Amine curing agents are the most prolific and have the widest application range among epoxy resin curing agents, as they account for 70% of all curing agents used. They mainly include aliphatic polyamines, aromatic polyamines, and the like. Although they are all amine-based curing agents, their different chemical structures cause various properties, curing speeds, and curing temperatures. The properties of their cured products also vary widely.

**Figure 5:**
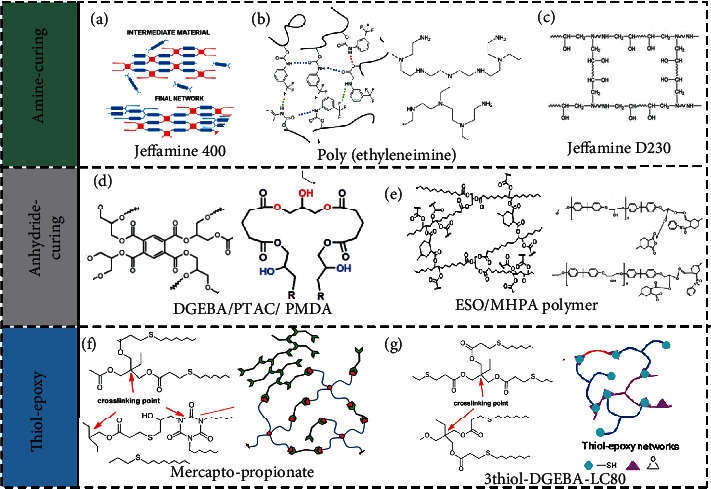
(a–c) Epoxy resin curing system: amine curing system. Reproduced from Ref. [51, 53, 56]. (d, e) Acidic anhydride curing system. Reproduced from Ref. [63, 70]. (f, g). Click on the chemical system. Reproduced from Ref. [71, 72, 74].

Polyetheramine contains flexible ether-bond groups, which can effectively improve the toughness of a product and also improve the mechanical properties of EP, but the curing speed is relatively slow. The molecular weight of polyetheramine can be regulated by various amine reagents such as D230 and D400. In 2009, Xie and Rousseau reported the curing of aromatic epoxy systems with Jeffamine D230 followed by adjustments of the addition of decylamine (DA) and neopentyl glycol diglycidyl ether (NGDE) [15], as shown in Figure 5(a). This was a facile method to precisely adjust the *T*_g_ of SMEP, ranging from room temperature to 89°C. Subsequently, they prepared a two-component epoxy-amine (E44/D230) SMEP with *T*_g_ adjustable between 40°C and 80°C and a fracture strain value of 212% at the *T*_g_ peak [50, 51]. Epoxy networks containing hyperbranched poly(ethyleneimine) polyetheramine crosslinks have been investigated in recent years, as shown in Figure 5(b). Morancho et al. [52, 53] studied the effect of hyperbranched structures on performance. The *T*_g_ of the system was adjusted from 60°C to 117°C as the content of hyperbranched poly(ethyleneimine) changed. The fracture stress and strain values of the material were significantly increased, which had potential for application to actuators [54, 55], as shown in Figure 5(c).

Konuray et al. [56] prepared a poly(hydroxylamine)-poly(ether) curing agent, so that the epoxy group can be cured twice intermittently. Liu et al. [57] prepared a series of SMEPs using epoxy resin 618 and different amounts of curing agent DDM. When the curing degree is 50%-100%, the *T*_g_ value of the system is 45°C-145°C. The elongation at break reaches the maximum when the temperature is 73.7°C. Similarly, Song et al. [58] also used DGEBA and DDM and added m-phenylenediamine (m-PDA) to achieve higher stiffness and *T*_trans_. Also, they investigated the effect of test temperature, curing agent type, and content on the viscoelastic behavior of these materials [59]. Feldkamp et al. reported that DGEBA was cured by a series of different amines, which increased the limit strain of EP by three to five times at different temperatures [60]. Furthermore, they studied the effect of chemical composition on shape memory properties based on the type and extent of curing agent added [61].

#### 2.2.2. Anhydride Curing SMEP System

Acid anhydride curing agents are used less frequently than amine curing agents. Anhydride-cured products have better dielectric properties than amine and are widely used in electrical insulation. Common anhydrides include phthalic anhydride (PA), methyltetrahydrophthalic anhydride (MHHPA), and hexahydrophthalic acid. Acid anhydrides require higher curing temperatures, but their low toxicity, low volatility, and ease of processing have attracted researchers' interest.

MHHPA is one of the most frequently used anhydride curing agents. Fan et al. [30] prepared two bisphenol A epoxy resins (DGEBAEO-2/DGEBAEO-6) containing ethylene oxide units, which were cured with HHPA. With the increase of DGEBAEO-6 content, the fracture strain increased, and the brittleness of the material improved. Liu et al. [62] used MHHPA to cure E-51 and added multiwalled carbon nanotubes (MWCNT) to prepare shape memory nanocomposites with high flexural modulus and maximum stress at room temperature. Tsujimoto et al. [63] used the MHHPA curing agent to treat biobased epoxy vegetable oil to obtain transparent and soft materials, which greatly reduced greenhouse gas emissions and became a renewable resource, as shown in Figure 5(e). Wu et al. [64] studied the optimization of shape memory effect (SME) in tetrahydrophthalic anhydride-cured EP, the effects of crosslink density, and programming temperature on SME.

Biju et al. synthesized carboxy telechelic poly(tetramethylene oxide) (PTAC) and reacted with an epoxy-anhydride system to obtain an SME [65, 66]. PTAC changes the kinetics of the reaction by interaction with epoxy groups. With the increase of PTAC content, the bending strength, modulus, and *T*_g_ of the system decrease. The shape fixation ratio and the recovery ratio of SMEP series are greater than 95%, as shown in Figure 5(d). Wei et al. [67] prepared SMEP using hydrogen epoxy resin, maleic anhydride, and polypropylene glycol diglycidyl ether (PPGDGE). The *T*_g_ of SMEP decreases from 110°C to 50°C, and the crosslink density decreases. Subsequently, they prepared a series of new SMEPs using hydrogen epoxy, MMHPA, and diglycidyl 4,5-epoxy tetrahydro phthalate (TDE-85) [68]. They then prepared a series of SMEPs with tetraposensitive epoxy monomer (AG-80) and glutaric anhydride [69]. With the increase of AG-80 content, the *T*_g_ and rubber modulus of the system increase, and the shape memory performance is excellent.

The direct reaction of functional groups to form dynamic covalent bonds has strong applicability and mainly exists in epoxy/anhydride systems. Dynamic transesterifications are usually carried out in an anhydride curing SMEP system. The related details are described in Section 2.4. Liu et al. [70] prepared epoxy glass ceramics using a glutaric anhydride-epoxy-glycerol system without a catalyst. However, the presence of glycerol led to a decrease in the crosslink density and *T*_g_ of the crosslinking network, demonstrating the potential applications in repairable coatings.

### 2.3. Thiol-Epoxy “Click” Systems

“Click chemistry” has the advantages of fast reaction speed, high selectivity, and mild reaction conditions, and the thiol-epoxy reaction has attracted much attention in recent years. The essence of the thiol-olefin click reaction is the addition reaction of thiol and a double bond. The mechanism includes a photo(thermal)-initiated free radical reaction and Michael-addition reaction to obtain functional polymers with controllable structure. Click chemistry is mainly used in polymer end-group modification to prepare hyperbranched polymers (HBPs) and photocurable materials.

Belmonte's group conducted extensive research in thiol-epoxy “click chemistry.” In 2015, they proposed thiol curing agents and epoxy resins to make a series of enhanced SMEPs with click chemistry. They studied the relationship between thermomechanical properties, network structure, and shape memory response [71]. Subsequently, they synthesized the epoxy resin and pentaerythritol (S4) via dual-curing technology and found that the uniform network structure could achieve a faster and narrower recovery process [72]. The network structure was designed according to the adjustable conditions, and the corresponding shape memory effect was predicted [73, 74]. In the above system, LCN with different thicknesses could change liquid crystal molecules' organization to prepare multilayer assembly materials [75]. Russo et al. [76] double-cured the mercaptan acrylate-epoxy resin system and characterized their rheological and mechanical properties, as shown in Figure 5(f). They adjusted the ratio of acrylate and thiol groups and combined the characteristics of the two networks to obtain a high-fracture final material with a colloidal intermediate state. Song et al. [77] prepared a biobased thiol-epoxy shape memory network formed from the gallic acid-based thiol and TDI glycidyl ether of bisphenol A (DGEBA). Besides, epoxidized vegetable oil is added to the system, which reduces the glass transition temperature and the tensile strength of the network and improves the toughening effect, as shown in Figure 5(g). Because mercaptan can overcome the oxygen polymerization inhibition reaction in photocuring, the photocuring reaction is dependent on mercaptan click chemistry. As an energy-saving and environment friendly curing method, UV curing is mainly divided into two mechanisms: free radical light curing and cationic light curing. Free radical photocuring is fast, but the curing depth is shallow, suitable for film formation. Cationic curing is not easy to terminate and has small shrinkage. It is suitable for curing three-dimensional parts. Wang et al. [78] used click chemistry to design the photoradical polymerization of epoxy resin and acrylate in ultraviolet light for secondary photocurability, which allows manufacturing a three-dimensional structure without mold.

### 2.4. Dynamic Covalent Bonding

Dynamic covalent chemistry makes irreparable crosslinked polymers possible. Dynamic covalent bond topological changes occur at high temperature, similar to conventional thermosetting resins. Crosslinked polymer materials are repeatable processing, self-healing, remoldable, and recyclable properties. Kloxin et al. [79] proposed that the bond exchange process of covalent adaptable networks can proceed via two mechanisms: “dissociative” and “associative” processes. The crosslinking of “dissociative exchange” undergoes two distinct breaking and re-forming steps, such as Diels-Alder (DA) addition. However, “associated exchange” belongs to a single-step exchange mechanism. Bond breaking and reforming occur simultaneously and constant crosslinking in the exchange process, such as transesterification and silicone ether. In particular, the “vitrimers” materials developed in recent years also belong to associative exchange reaction, making the crosslinking network in permanent but dynamic crosslinks and maintaining high crosslinking density [80, 81]. Zheng et al. [82] comprehensively reviewed various types of dynamic covalent bonds from the molecular design perspective. Also, they summarized the effects of different dynamic covalent bonds on the performance of SMPs. In addition, Zhang et al. [83] divided them into two categories according to the reactants before and after dynamic reaction. One is a dynamic reversible covalent exchange reaction. Another reversible covalent reaction includes reversible addition and reversible condensation.

**Figure 6:**
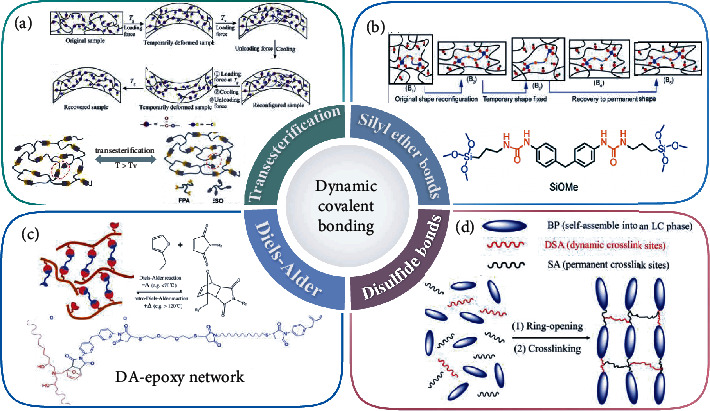
(a) Transesterification reaction. Reproduced from Ref. [85, 87]. (b) There is siloxane exchange. Reproduced from Ref. [88]. (c) DA exchange. Reproduced from Ref. [89]. (d) Disulfide exchange bonds. Reproduced from Ref. [90].

In 2011, Montarnal et al. [84] found that epoxy resin and acid anhydride crosslinking had rheological properties similar to glass based on their dynamic covalent bond network and proposed a new concept of “vitrimer” for the first time. Ding et al. [85] synthesized SMEPs with new high-performance thermosetting properties based on dynamic ester exchange bond of EP/CBMI system. Li et al. [86] introduced the transesterification of esters and hydroxyl groups in the liquid crystal epoxy system to form polymers that could be reshaped and repaired. Different functional blocks allowed the system to have 2W-SMP, self-repair, and processability. Epoxidized soybean oil (ESO) is an excellent biobased vitrimer resin that can react quickly with other compounds containing carboxyl or anhydride groups. However, the high flexibility of the ESO chain results in *T*_g_ close to room temperature and poor mechanical properties as shown in Figure 6(a). Yang et al. [87] introduced resin derivatives based on vitrimer resin to improve mechanical properties and *T*_g_. Since the rotation or torsion of the backbone bonds of rosin derivatives was restricted, they exhibited rigid properties. In addition, Song et al. [77] used ESO and vanillin to synthesize very strong polymers. The doubly dynamic crosslinked network of hydrogen bonds and dynamic imine bonds in the system enabled the damaged polymer to heal itself and be recycled multiple times.

In addition to the transesterification reaction, there are siloxane, DA, and hydrogen-bonding reactions. Silyl ether is a dynamic covalent bond with good thermal stability and robust strength. The crosslinking network is adjustable and has a high *T*_g_. Ding et al. [88] adjusted the *T*_g_ of SMEP from 118.1°C to 156.4°C, which showed higher tensile strength. The material could also be transformed from a flat film to a crossinked network of dynamic silyl ether bonds of various shapes with high toughness, as shown in Figure 6(b). Yang et al. determined that as the end-to-end distance of the polymer chain decreased, the DA network exhibited higher flexibility, as shown in Figure 6(c) [89]. Li et al. [90] synthesized a liquid crystalline epoxy network with exchangeable disulfide bonds. The rapid disulfide exchange reaction rearranged the network structure, as shown in Figure 6(d).

## 3. Multifunctional Shape Memory Epoxies

### 3.1. Multiple Responsive SMEPs

The stimulus-responsive method of SMPs has gradually evolved from a single-heating driving method to various new driving methods using light, electric field, electromagnetic field, radiowave, or solvent. As research has progressed, it has become apparent that these driving methods still need to be improved to meet the broader application requirements of SMPs. In the past, multiresponsive SMPs were achieved through traditional physical doping of functional particles. The introduction of functional groups into crosslinked networks to achieve multifunctional integration of SMPs gradually became a new trend.

**Figure 7:**
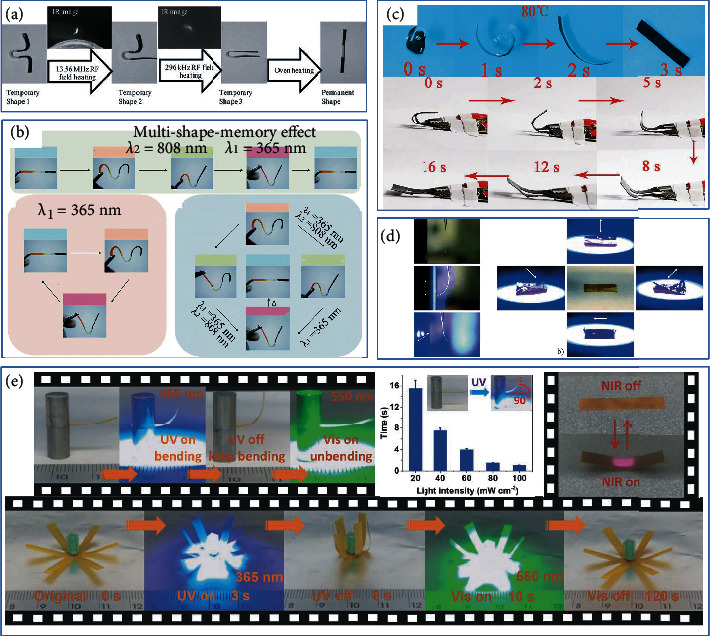
(a) Selective heating shape memory recovery in different RF fields. Reproduced from Ref. [96]. (b) Selective heating shape memory recovery of regions at different light wavelengths. Reproduced from Ref. [97]. (c) SMEP completes the shape memory process quickly under voltage and NIR. Reproduced from Ref. [100]. (d) Demonstration of thermal and UV-induced shape memory behavior of LCEN. Reproduced from Ref. [102]. (e) SMEP responsive to NIR and ultraviolet light (UV). Reproduced from Ref. [103].

There are situations where it is not easy to achieve direct heating. Under certain conditions, especially in aerospace structures, it is convenient and fast to use an electrical heating to trigger the SME [91–94]. Carbon black (CB) has a good photothermal conversion effect on the infrared laser. It can selectively irradiate specific parts of the material to achieve deformation. Liu et al. filled the hydrogen epoxy resin (HEP) with different amounts of CB to obtain laser-triggered SMPCs [95]. He's group has prepared SMEP composites with selective radiofrequency actuation [96]. They combined Fe_3_O_4_, MWCNT, and pure SMEP to obtain three-stage multiple responsive SMEPs. In addition, different response frequencies make the system have remote-controllable selective shape recovery performance, as shown in Figure 7(a). Yang's research team also prepared a SMEP multiresponsive composite, which selectively locally restored the shape through different light wavelengths [97, 98], as shown in Figure 7(b).

The addition of graphene to the polymer yields more excellent properties for the resulting composite. Zhang et al. [99] added graphene to polyurethane/epoxy (PU/EP) composite materials to achieve dual thermoelectric response characteristics. Shape memory performance was excellent due to interpenetrating network structure (IPN). Wang et al. [100] prepared a novel of reduced graphene oxide (RGO)/waterborne epoxy (WEP)/RGO sandwich structure composite membrane. RGO paper had excellent conductivity and thermal conductivity. Samples quickly complete the shape memory process under voltage and near infrared (NIR) irradiation. Lamm et al. [101] used supramolecular soybean epoxy resin and cellulose nanocrystals to synthesize heat and chemical responsive SMEPs. Using solvents that can destroy hydrogen bonds, hydrogen bond destruction can have chemical reaction behavior. Li et al. [102] combined thermally responsive liquid crystal, light-responsive azobenzene molecules, and dynamic disulfide bonds to form a multifunctional integrated LCEN. Under UV light or thermal stimulation, dynamic disulfide bonds make the system repairable and recoverable. Lu et al. [103] doped gold nanorods into an azobenzene liquid crystal network to form a composite material that can respond to two different types of light. Due to the photochemical reaction of azobenzene and the photothermal effect on the surface of AuNRs, the composite materials have NIR and ultraviolet light (UV) responses.

### 3.2. Multishape Memory SMEPs

A triple-shape SMP (TSMP) based on epoxy resin can the two internal networks are semi-interpenetrating networks and full interpenetrating networks. On the other hand, two resin layers with different *T*_g_ are laminated to obtain TSMP in the traditional sense of the composite system. TSMP is characterized by a wide *T*_g_ range or multiple *T*_trans_ [104, 105]. The number of temporary shapes that can be memorized in SMPs is directly related to the number of discrete reversible phase transitions in the polymer. TSME regulation can be achieved by adjusting the shape memory transition temperature, which requires changing the composition of the system. The keys to a triple-shape SMP are as follows: (a) having a wide *T*_g_ range or generating a universal network with two “transitions”, or (b) combining two different *T*_g_-EP layer systems [106].

**Figure 8:**
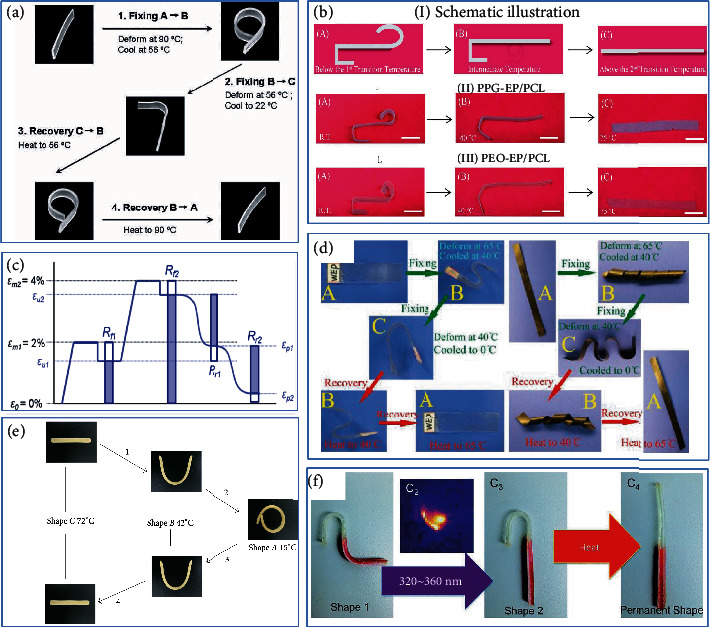
Process diagram of EP with multiple shape memory. (a) The double-layer epoxy resin shows TSME. Reproduced from Ref. [105]. (b) Triple-shape memory composites prepared by polymerization-induced phase separation. Reproduced from Ref. [42]. (c) Schematic diagram of triple shape memory test. Reproduced from Ref. [107]. (d) TSME of CNT/WEP nanocomposites. Reproduced from Ref. [109]. (e) SMEP composite bilayers with TSME. Reproduced from Ref. [109]. (f) UV and thermal remote dual trigger shape recovery SMEP. Reproduced from Ref. [110].

Xie's group has conducted an in-depth research on triple-SME (TSME). They combined two SMEPs that exhibited different *T*_g_ into a two-layer polymer and adjusted the shape fixing of the TSME by changing the ratio [105], as shown in Figure 8(a). Torbati et al. [42] used polymerization-induced phase separation (PIPS) to generate microstructures. Compared to the corresponding PCL, poly(*ω*-pentadenolactone) (PPDL) produces higher crystallinity, as shown in Figure 8(b). Fej et al. [107] prepared a triple-shape memory system of EP/PCL and designed its temporary shape based on the *T*_g_ of EP and the melting temperature (*T*_m_) of PCL. Arnebold et al. formed an EP/PPDL heteromorphism via segregation and crystallization to improve the strength and toughness of materials. The rapid shape fixation and good shape memory cycle stability took approximately 30 s [108]. Ordinary CNT/SMEP composites can only remember the dual shape memory effect of a temporary shape. Dong et al. [109] introduced bisphenol A-toluene dissocyanate-Triton X100 (EP-g-TX100) as the reactive emulsifier. EP-g-TX100 has a good emulsifying ability and dispersing ability to disperse CNTs. Subsequently, they mixed various nanosilica particles with different contents to prepare double-layered SMEP composites [109]. A three-layered SMEP of the nanocomposite layer was achieved, and the mechanical properties and shape memory properties of the materials were significantly improved.

In addition to the methods above, Wu et al. [110] developed a dual response to achieve selective local triple-SMEPs that remotely triggered shape recovery through UV light and heat, as shown in Figure 8(f). Since Zn(Mebip)_2_(NT*f*_2_)_2_ has the characteristic of converting UV light into heat, the TSME is realized under the condition of SMEP without the need for a wide shape memory *T*_trans_ range and two regions containing two different shape memories.

### 3.3. One- and Two-Way SMEPs

Common SMPs exhibit a one-way shape memory effect (1W-SME), which is irreversible. However, some polymers such as LCE, IPNs, and double-layer structures exhibit a bidirectional two-way shape memory effect (2W-SME). The characteristics of reversible conversion can make the material have broader application prospects and higher practical value.

**Figure 9:**
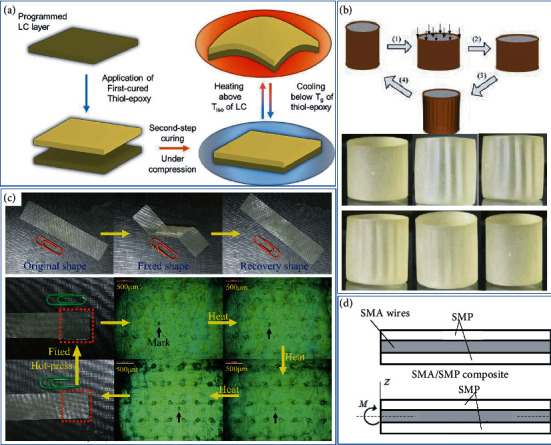
Process diagram of bidirectional shape memory epoxy resin. (a) LCN-EP composite layer programmed bidirectional shape memory. Reproduced from Ref. [111]. (b) 2W gradient SMEP column system. Reproduced from Ref. [112]. (c) WEP films exhibit bidirectional SMEP at low heating rate. Reproduced from Ref. [113]. (d) 2W bending-driven NiTi wire and epoxy-based SMA/SMP composites. Reproduced from Ref. [115].

LCNs have a reversible isotropic LC transition, which can reversibly expand and reversibly shrink during heating and cooling, yielding a true two-way SMP. Belmonte et al. [111] used a “thiol-epoxy” mixture system for dual curing to obtain a glassy thermosetting (GT) film and a programmed LCN to form a multilayer structure. LCN- EP layer was “programmed” under stress before being inserted between the two thiol cured EP layers as shown in Figure 9(a). They also used different aliphatic chain long carboxylic acids to cure epoxy-based liquid crystals. With the change of the aliphatic chain length of the curing agent, the liquid crystal degree also changed, and the isotropic temperature higher than 100°C and the LCN with high driving stress and strain were obtained. The length of the aliphatic chain and the programming conditions were balance, and a controlled and stable driver can fine-tune the LCN. Wang et al. [112] formed a bidirectional gradually changing shape memory system by surrounding the SMP film with high *T*_g_ on another SMP cylindrical core with low *T*_g_. Dong et al. [113] pressed the powder of WEP and curing agent into a thin film, and laid two layers of release fabric to prepare EP, under the freeze-drying and hot-pressing techniques. The system shows unidirectional SME phenomenon and bidirectional SME phenomenon at a low heating rate.

Controlled behavior composites (CBCM) are generally deformed thermoactive composites with asymmetric bimetallic ribbon effects. Basit et al. [114] formed a seven-layer CBCM composite board with epoxy resin to form a 2W-SMEP. Under different recovery temperatures, the one-step programming system shows a two-way shape memory effect in the process of unconstrained recovery. Similarly, Taya et al. [115] made NiTi wire and epoxy-based SMA/SMP composites capable of bidirectional flexural drive, as shown in Figure 9(d). During the heating and cooling process, the SMA wire showed a large two-way deflection, in which the SMEP matrix provided a biasing force. At the end of the cooling process, the SMP could fix the shape without additional energy.

## 4. SMEP Composites

Shape memory polymer composites (SMPCs) have the advantages of large recovery strain, high reliability, low density, high rigidity, and high strength, which are beneficial to different structural applications. In particular, the lightweight deployable spacecraft structures have great potential, which exhibit an SME and have multiple responses to stimuli. EP has the advantages of good mechanical properties, low water absorption, high-temperature resistance, a low curing shrinkage, and low thermal expansion coefficient. Therefore, in the current practical applications, SMEP composites structures are applied more often.

To date, the following SMPs have been discovered: polyurethane (PU), polyimide (PI), polystyrene, cyanate ester resin (CE), epoxy resin (EP), and bismaleimide resin (BMI) [116–120]. The properties of the above materials are shown in Table 1. In summary, the EP is a SMP with excellent comprehensive performance, followed in performance by polyester and polyurethane.

The most prominent studies in EP include TEMBO® series of CTD (composite technology development) and the TP series of ILR Dover. The TEMBO® series of SMEP is used as a matrix and is compounded with reinforcing fibers to design the expanded structure of the solar cell arrays [136]. ILC Dover has been studying several SMPs [137]. In 2007, CRG of the United States introduced carbon fiber-reinforced epoxy shape memory composites to the market [138]. Their products are in a leading position in the field with adjustable *T*_*g*_ values and high modulus of elasticity and strength over a wide range of temperatures above 0°C.

### 4.1. Toughening Modification of SMEP Composites

The conventional epoxy resin crosslinking network has a short molecular chain length between the crosslinking points and a large rigidity of the molecular chain. Even above *T*_g_, it exhibits significant brittleness, and its elongation at break is still very limited, which significantly limits the application of the material in the field of shape memory materials. Therefore, an increasing number of researchers are studying the toughening of epoxy resins. At present, epoxy resin toughening technology mainly includes flexible chain toughening, rubber mixed toughening, core-shell structure toughening, thermotropic liquid crystal polymer toughening, IPN toughening, and hyperbranched polymer (HBP) toughening.

Flexible chain toughened epoxy resins are bonded to the epoxy resin crosslinked network to form a tight and loose interphase network structure. Wang et al. [95] crosslinked epoxy resin diglycidyl ether of bisphenol F (DGEBF) with toughening agent 3-ethyl3-oxyheterocyclobutanol (TMPO), providing the possibility for 3D printable SMEP. To improve the toughness of the epoxy/anhydride system, Fan et al. [30] bonded different amounts of oxyethylene units to epoxy resins and cured them with hexahydrophthalic anhydride. With the increase in DGEBAEO-6 concentration, the *T*_g_ and storage modulus of SMEP gradually decreased.

In the toughening system of rubber elastomer, the formation of dispersed phase will lead to stress concentration, to promote the dispersion of external action energy. The reactive rubber elastomers mainly include carboxy-terminal nitrile rubber (CTBN), amino-terminated nitrile rubber (ATBN), hydroxyl-terminated polybutadiene rubber (HTPB), and polyacrylate rubber. Li et al. modified the E-51/MeTHPA shape memory epoxy resin with CTBN [139]. The rubber particles absorbed external impact stress, the toughness and shape recovery ability of the material were improved, and the tensile strength was reduced. Wei et al. [95] added crosslinked carboxynitrile butadiene nanorubber (CNBNR) to the SMEP system to improve overall performance. When the CNBNR content reached 20 phr, the elongation at break increased from 49.5% to 736.4%, which greatly improved the elasticity of the SMEP composite. Similarly, Revathi et al. added CTBN to SMEP resulting in a slight decrease in storage modulus and *T*_g_ [140], with better durability and shape recovery performance than unmodified SMEP.

Amorphous or semicrystalline thermoplastic resins have roughly the same toughening mechanism as rubber but hardly affect mechanical and thermal properties. Lützen et al. [43, 141] copolymerized polymerized hydroxyl-terminated semi-crystalline PCL with EP to form a conetwork. Subsequently, the researchers proposed a fast, switchable SMEP that was polymerized with high-melting cations and low-melting polymers [108]. When the weight of PPDL was 10%, the tensile strength of the sample reached the highest value of 33 ± 3 MPa. Chen et al. [142] used direct ink writing (DIW) 3D printing to produce high tensile toughness thermally cured epoxy composites. The second stage of thermal curing produces toughened IPN composites. Yao et al. [143] used polyurethane PU to construct a foam skeleton and grafted EP into the PU network to make a shape memory composite foam. Multiple crosslinked networks are entangled to form IPN.

HBP is similar to extended dendrimers in that they have low molecular weight as a growth site and gradually control the molecular weight of repeated reactions. Wang et al. [144] synthesized a hyperbranched polyurethane (HBPU) with triazine structure grafted onto an epoxy resin (EP) to form IPN of different proportions of HBPU/EP. IPN composite with HBPU content of 20 wt % had the best shape fixation and recovery. Santiago et al. [145] used hyperbranched polymers and aliphatic diamine-modified epoxy shape memory polymers. The hardness and impact strength of the system decreased, and the tensile property increased with the increase of hyperbranched polymer content.

Core-shell structure gauging mainly controls the effect by controlling particle size, the number of shell layers and distribution uniformity. Zhang et al. [97] prepared a PCL/EP composite fiber with a core/shell structure system. The electrospun composite fiber can improve mechanical strength and elongation at break. Neuser et al. [146] encapsulated amine curing agents and acrylate as microcapsules to form an epoxy/amine self-healing system. A rough fracture surface of the sample was observed, and the sample showed high fracture toughness after healing.

**Figure 10:**
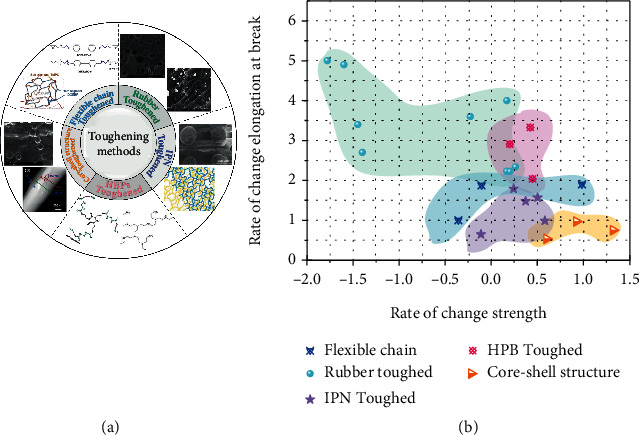
(a) Different toughening methods of SMEP composites. Reproduced from Ref. [30, 95, 97, 140, 142–146]. (b) Comparison of toughening effect of SMEP composites.

For the above toughening means, the rubber toughening effect is best, but strength takes a loss. Although the strength can be greatly improved for core-shell toughening, the toughening effect is general. HPB toughening reduced chain segment entanglement and improved crosslinking network density to obtain a tough and strong system, as shown in Figure 10(b).

### 4.2. Strengthening the Mechanics of SMEP Composites

**Figure 11:**
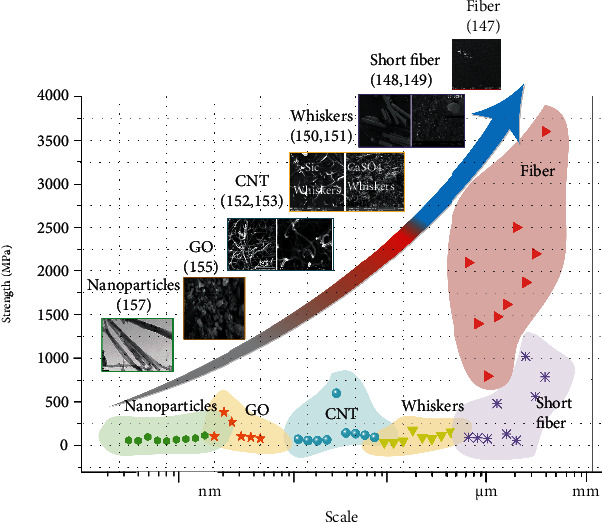
Comparison of mechanical properties of SMEP composites with different reinforcements. Reproduced from Ref. [147–150, 152, 153, 155, 157].

Reinforcement of SMEP composites by continuous fibers, dispersed particles, whiskers, etc., can significantly improve the material's mechanical properties such as strength, stiffness, relaxation, and creep. The reinforcement may include carbon fibers, glass fibers, spandex fibers, carbon nanotubes, silicon carbide whiskers, and POSS nanoparticles, as shown in Figure 11.

Liu et al. [147] added various short and continuous carbon fibers (CF) to pure shape memory epoxy resin matrix. The storage modulus of SMPC at room temperature and *T*_g_ are as high as 37 GPa and 4.4 GPa, respectively, showing excellent mechanical properties. Herath et al. [148] formed 0/90° woven carbon fibers made of prepreg into shape memory epoxy composites. The structural properties of carbon fiber-reinforced SMPC were significantly improved, and the prepreg can be widely used in large-scale engineering applications. Wei et al. [149] used chopped glass fibers to enhance shape memory hydroepoxy composites. With the increase of short glass fiber content, the glass modulus and flexural strength of the material increase, and the content slightly decreases after 6%. The *T*_g_ value of the system has almost no effect. Dong et al. [150] prepared a vapor-grown carbon nanofiber (VGCNF)/SMEP nanocomposite by latex technology. Latex technology is relatively simple, versatile, repeatable, and reliable and does not require organic solvents. Carbon nanofiber-reinforced epoxy resins have significantly improved mechanical properties.

Likitaporn et al. [151] prepared benzoxazine-epoxy resin shape memory composites by filling silicon carbide (SiCws) whiskers with 0-20 wt%. The storage modulus at *T*_m_ increased with the increase of silicon carbide whiskers, from 5.1 GPa to 8.8 GPa, and the *T*_g_ also increased by nearly 20°C. Wang et al. [152] introduced different amounts of SiCws into the EP matrix to form SMEPC. When the content reached 12 wt%, the bending strength of the material increased by 64.1%, and the *T*_g_ value decreased slightly with the increase of SiCw content. Subsequently, they investigated the effects of calcium sulfate whiskers (CSW) on the thermodynamic and shape memory properties of epoxy/cyanate SMP composites [153]. When the content of CSW is 5 wt%, the flexural strength was improved by 29% compared with the pure resin. Liu et al. [147] added graphene oxide (GO) to SMEP. When the GO content was 0.8 wt%, SMEPC had good shape memory properties and the best thermal and mechanical properties.

There are many highly active unpaired atoms on the surface of the rigid inorganic nanoparticles, which are conducive to the group reactions in the epoxy resin to improve the interfacial binding force and achieve strengthening. Revathi et al. [140] used CNTs to enhance the shape memory of epoxy nanocomposites. At a higher deformation temperature, the reinforcement effect of carbon nanotubes is better and more obvious. Abishera et al. [154] proposed a study on the reversible plastic shape memory (RPSM) properties of multiwalled carbon nanotube- (MWCNT-) reinforced epoxy nanocomposites. They then investigated the superior properties of the system under bending and torsional deformation [155]. Yun and Liang [156] prepared a series of shape memory epoxy composites reinforced with different levels of carbon black (CB). The results showed that the addition of CB particles significantly increased the stiffness of the SMEP and reduced the viscoelasticity of the system. Similarly, Wei et al. [157] studied the electroactive shape memory water-epoxy/carbon black composite. They also prepared a series of organic-inorganic hybrid resin systems using epoxy-functional polyhedral oligomeric silsesquioxanes (POSS-EP) [158]. With the increase of the POSS-EP, the flexural strength increased first and then decreased, indicating that the POSS-EP content of 3.17 mol% was the extreme value of the hybrid material. Wang et al. [159] mixed GO and carbon nanotubes (CNT) into WEP to prepare a ternary hybrid polymer shape memory composite. GO effectively dispersed CNT to make both of them evenly dispersed in the WEP matrix, significantly improving the mechanical properties, thermal conductivity, and thermal response speed of GO/CNT/WEP composites.

### 4.3. Extreme Environmental Resistant of SMEP Composites

With increasing applications in the fields of aeronautics and astronautics, there are more requirements for operating conditions and more stringent requirements for performance. Spacecraft in orbit operation is mainly affected by many environmental factors, including high vacuum, high and low-temperature cycle, charged particle irradiation, vacuum ultraviolet irradiation, and atomic oxygen. Therefore, improving the space environment resistance of SMEP is very important to expand the scope of application.

The polar hydroxyl groups in the epoxy resin lead to poor resistance to high temperatures. At present, the heat resistance of epoxy resins is improved mainly by mixing the polymer with high *T*_g_ materials, increasing the crosslinking degree of the resin, or forming interpenetrating networks. Silicon-oxygen bonds have high bond dissociation energy, are not easily broken, and exhibit excellent high-temperature stability. Ding et al. [88] used 3-isocyanatopropyltrimethoxysilane (EPSis) and EP to make the dynamic covalent bonds of silicone ethers, which exhibited a high *T*_g_ and a change in *T*_g_ from 118.1°C to 156.4°C. Zhang et al. [153] prepared a series of PEO-POSS systems using POSS-terminated polyethylene oxide. The formation of POSS microregion enhances the strength of the material. When the content of POSS is 10%, *T*_g_ can reach about 150°C. High-temperature-resistant resins generally result from the inclusion of special functional groups, such as polyimide (PI), maleimide (BMI), and cyanate (CE). Their *T*_trans_ is much higher than those of epoxy resins, and the combination can improve the high temperature resistance of epoxy resin. Rimdusit et al. [40] mixed BA-a benzoxazine monomers into epoxy resins. The molecular rigidity of benzoxazine improves the heat resistance of the network, and the samples with the highest *T*_g_ increase by nearly 70°C. Subsequently, they filled 0-20 wt% high adamantane silicon carbide whiskers on this basis, and the *T*_g_ was increased to 170°C [151]. Ding et al. [85] added different concentrations of chain-expanding bismaleimide resins (CBMI) to SMEPs and obtained a series of EP/CBMI polymers with high *T*_g_. Details have been introduced in previous chapters as shown in Section 2.1.2. Because there are a small number of open-loop and crosslinked epoxy functional groups in the curing process, two-stage curing can be thermal-curing or photocuring to improve the degree of crosslinking [160]. Liu's group proposed that secondary discontinuous solidification and two independent stages could control the degree of crosslinking to realize the simultaneous demand of being soft and hard in different environments [161]. After two-stage curing, *T*_g_ increased from 84°C to 130°C, and the strength was also improved.

Radiation resistance and high and low temperatures in space can be extremely damaging to materials. Polymer will produce irradiation crosslinking and irradiation degradation at the same time under irradiation, which restrict each other. Because the epoxy resin contains more stable groups, it has certain radiation resistance, but its performance will decline after exceeding a certain dose. *γ*-ray is a high-energy ray with enough energy to destroy, such as C-C bon and C-O bond. Leng et al. [122] evaluated the thermal and mechanical properties of SMEP under *γ* irradiation of 1 × 10^6^ Gy. The mechanical properties and shape memory properties of the material remain excellent because the chemical bonds in the system do not change before and after irradiation. Jang et al. [162] tested the space environmental properties of carbon fiber-reinforced SMEP composites with an amine curing system. Under the exposure of high vacuum and ultraviolet radiation, ultraviolet has a crosslinking effect on the free radicals not involved in the reaction in the main chain of SMP and predicted the long-term life performance in this environment. The space environment temperature changes alternately. The sunny side absorbs the radiant heat of the sun, and the temperature is more than 100°C, while the sunny side is less than -100°C, which forms a very high and very low uneven temperature environment. During the cooling process, even under ultralow temperature environment, EP will produce thermal stress due to thermal shrinkage. When the thermal stress exceeds its strength, the material will be damaged. The ultralow temperature properties of epoxy resins can be improved utilizing toughening, such as adding flexible aliphatic resins and thermoplastic plastics. Tan et al. [163] conducted a thermal cycle test after 45 cycles at high and low temperatures (-100°C ~100°C). Due to the high temperature, the postcuring of epoxy resin increased the crosslinking density and improved the mechanical properties.

## 5. Application

### 5.1. Aerospace Applications

**Figure 12:**
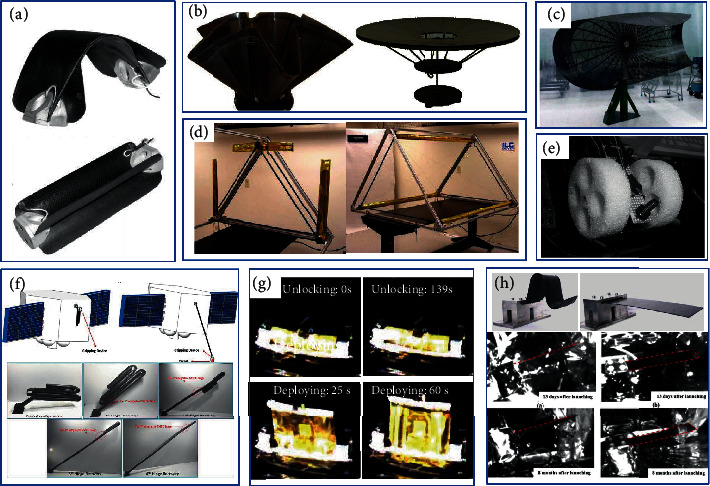
(a, b) Lightweight EMC hinge of carbon fiber-reinforced composites. Reproduced from Ref. [176]. (c) Open carbon fiber/EP laminate for Taco Shell reflector. Reproduced from Ref. [158]. (d) Truss in both the deployed and packed positions. Reproduced from Ref. [166]. (e) SMP foam actuator. Reproduced from Ref. [170]. (f) Rigid solar wing based on shape memory hinge. Reproduced from Ref. [174]. (g) Recovery process of the LCCT. Reproduced from Ref. [175]. (h) On-orbit verification of SMEPC. Reproduced from Ref. [173].

Most aerospace composite materials use thermosetting resins because they have better mechanical properties, processing flexibility, temperature capabilities, and environmental durability. In particularly, epoxy resins show excellent shape memory characteristics and excellent rigidity and strength. The SMEPs used in space so far have been reported to include ILC Dover, Inc. and CTD. CTD designs a light space expansion truss composed of cylindrical tubes similar to antenna support tubes [164]. They also developed SMEP composite materials for large-capacity high-frequency reflectors for satellite communications [165], as shown in Figure 12(b). ILC Dover combined spring steel and SMEP tubes to form a dish-shaped parabolic space expansion reflector with a large compression ratio and good folding effect [166], as shown in Figure 12(d). The US Air Force Laboratory has developed a new satellite called RoadRunner. Its lightweight solar cell array will use carbon fiber-reinforced SMEP composite hinges [167]. The hinge device has the advantages of lightweight, simplicity, low thermal expansion coefficient, small vibration, and controllable deployment.

Compared with shape memory alloys, SMPs have excellent strain during heated, opening up their application in aerospace structures. However, the stiffness of the polymer can be improved by adding reinforcement materials [168]. Guo et al. [25] prepared shape memory liquid crystal epoxy composites based on glass fibers and nanosilica and used as new candidate materials for aerospace. Shape memory polymer foam (SMPF) is another potential field of shape memory technology. The main advantage of this material is a large amount of compression at the transition temperature. It can be applied to support structures in deployable spaces, shelters for shelters, and rover components. Compressed SMP in an open honeycomb foam can be used to build structures with various shapes, ranging from biomedical uses devices (such as embolization sponges) to advanced fuselage wings. CTD company has developed a SMPF that has been used in the aerospace industry [169]. Fabrizio et al. [170] produced samples through solid foaming and conducted multiple recovery tests to design foam actuators for space applications, as shown in Figure 12(e). In the last flight of the space shuttle Endeavour, SMEP foam prototype was selected for the international space station experiment.

Leng et al. [121] developed thermosetting SMEPs and tested the performance of the polymers and their composites against spatial extreme spatial (temperature, irradiation and vacuum). In addition, CB, CNTs, and chopped carbon fiber were added to SMEP to prepare a SMEP composite with conductive properties [23–25]. The results showed that the addition of CB, CNTs, and carbon fiber gave the composites electrical conductivity; Simultaneously, the mechanical properties of the material were also enhanced. Compared with the traditional SMPC hinge, the integral hinge [171] made of carbon fiber-reinforced SMEP composites had higher reliability and higher postdeployment stiffness and strength characteristics. Since the material level on-orbit verification of shape memory composites was realized in 2016, the ground verification of rigid solar wing based on shape memory hinge was carried out in 2018, and the on-orbit verification of flexible solar wing based on shape memory pod rod was carried out in 2020 [172–175], as shown in Figures 12(f)–12(h).

### 5.2. 4D-Printed Structures

**Figure 13:**
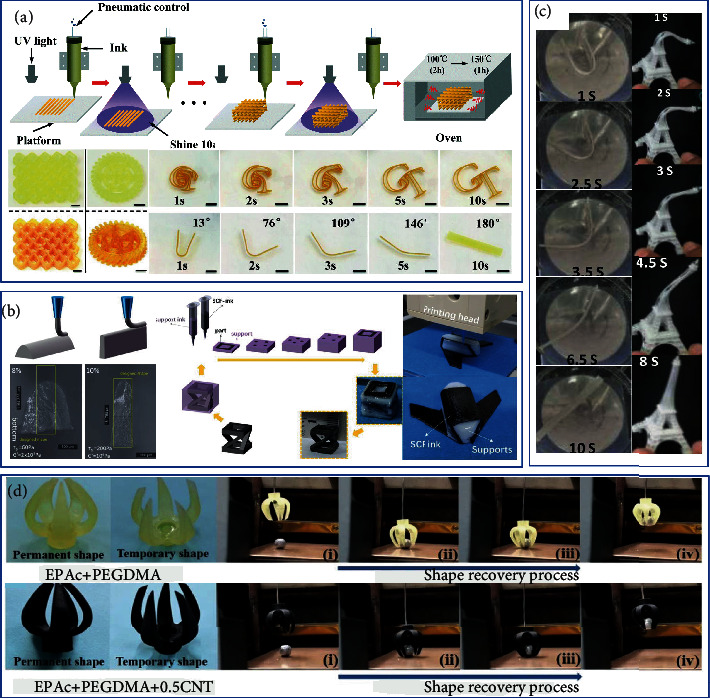
(a) Schematic illustration of the 3D printing epoxy composites. Reproduced from Ref. [142]. (b) Sketches and optical images of printed vertical wall. Reproduced from Ref. [178]. (c) Shape recovery process of the printed Eiffel Tower. Reproduced from Ref. [180]. (d) 4D printed gripper structure. Reproduced from Ref. [181].

Three-dimensional (3D) printing is an advanced manufacturing method. When the 3D printing system encounters SMP materials, it becomes 4D printing. In recent years, 4D printing has demonstrated unparalleled flexibility in the manufacture of complex three-dimensional structures and has received wide attention. The design of 4D printing structures brings endless ideas for applications such as biology, architecture, and smart devices. Under the 3D printers, shape design will play a more critical role in SMP/SMPC applications. Combining SMP/SMPCs with 3D printing provides excellent opportunities for soft robots, flexible electronics, and medical devices. Generally, there are many thermoplastic polymers in printing materials, and there are few studies on chemically crosslinked thermosetting resins [177]. Direct ink writing (DIW) has attracted attention due to its more selective printing materials and lower cost. Chen et al. [142] used UV-assisted DIW technology to photocure resins and improve mechanical properties through secondary thermal curing, as shown in Figure 13(a). The choice of thermosetting resins and nanoparticles can expand the range of 3D printing and directly print thermosetting materials with adjustable properties for high-performance and functional applications. Guo et al. [178] prepared epoxy-based ink-fumed silica for DIW as a rheology modifier and enhanced phase printing samples showing higher printing resolution. In addition, precise SLA 3D printing technology is cured by liquid photopolymer under UV irradiation, as shown in Figure 13(b). Honeycomb structures with layered pores fabricated by 3D printing show very high mechanical strength that cannot be achieved by traditional manufacturing processes. [179]. Yu et al. [180] found that dual curing of the system formed an IPN structure, and the glass rubber modulus of the printed sample was greatly improved, as shown in Figure 13(c). Wang et al. [181] printed claw catcher devices using photocured photosensitive composite ink, which was expected to be used in aerospace, such as grasping spacecraft or explosive debris with service termination, as shown in Figure 13(d).

### 5.3. Self-Healing of Cracks

**Figure 14:**
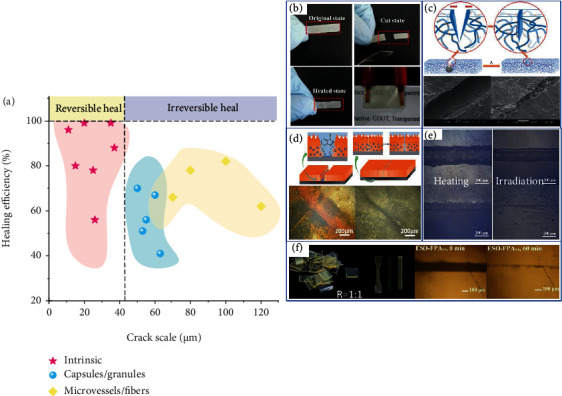
(a) Rrelationship between different repair modes and crack size and repair efficiency. (b) Self-healing SMEP with dual thermal and electrical response. Reproduced from Ref. [184]. (c) Schematic diagram of shape memory assisted self-healing coating concept. Reproduced from Ref. [185]. (d) SMEP scratch repair with CB photothermal filler. Reproduced from Ref. [186]. (e) Optical image of self-healing behavior of SMEP coating. Reproduced from Ref. [187]. (f) Transesterification reaction realizes self-healing and degradation and can be recycled. Reproduced from Ref. [189].

In harsh environments, microcracks may form inside the material, and fatigue damage may eventually damage the material. Thus, self-healing materials have become particularly important. Combined with the shape memory performance, the release force of the recovery is used to accelerate the crack repair process. Introducing different self-healing systems into polymer matrix can repair cracks of different sizes. Microvascular self-healing is mainly suitable for repairing large-scale crack damage, which belongs to microrepair. Internal self-healing is more suitable for repairing microcracks, which belongs to molecular repair. The self-healing crack size of the microcapsule is between microvascular self-healing and internal self-healing, as shown in Figure 14(a).

Microvessel and microcapsule are irreversible repairs, which belong to external repairs and are suitable for repairing large cracks. EMSP/PCL composites also have excellent SMP effects. Karger-Kocsis [182] determined that the repair efficiency also depends on the repair temperature, and the healing efficiency value is 50-70%. Luo et al. [183] proposed a self-healing AgNW/SMP composite with different stimuli, which can realize crack repair of tens of microns. Wei et al. [184] dispersed PCL particles in the EP matrix to produce shape memory and self-healing polymers, with a repair efficiency of 78.4%, as shown in Figure 14(b). Luo and Mather [185] proposed a new shape memory-assisted self-healing (SMASH) coating, as shown in Figure 14(c). Chen et al. [186] used carbon black (CB) as a photothermal filler to heal scratches at high temperatures, which could also repair scratches via NIR light in a remote manner. The diffusion and rearrangement of molecular chains in the healing area were observed as shown in Figure 14(d). Dong et al. used polydopamine@polypyrrole nanoparticles as photothermal agents to make thermally responsive SMEPs [187], as shown in Figure 14(e). Corrosion products present inside the scratch prevented the SME from closing. Therefore, the self-healing ability was introduced into the superhydrophobic coating to repair the damaged surface morphology, which could extend the service life of the material. Repairing wide-scale cracks, large cracks, and defects is a great challenge. The addition of meltable thermoplastic improved fluidity and increased the diffusion distance. When the original crack gap was about 50 *μ*m and gradually heals. Li et al. [188] used strain hardening by cold-drawing program to increase the restoring stress of SMP on both sides of the crack, which helped to heal the original 0.15 mm wide crack in the crack-closing matrix to 60-20 *μ*m.

Intrinsic self-healing is realized by entering a dynamic covalent bond, which reversible repair. It is suitable for small microcracks but can be repaired many times. However, the introduction of dynamic covalent bonds can make the material self-healing and recyclable. Vitrimers are covalent polymer networks that react to the topology of the network through bond exchange. Yang et al. [87] prepared biobased epoxy vitrification. Transesterification is used to realize self-healing at high temperatures, ethanol degradation, and recovery, and it can be degraded without new catalyst. Wu et al. [189] used ESO and natural glycyrrhizic acid (GL) to make biobased recyclable vitrimers, which showed excellent mechanical properties and thermal stability. Crack widths of 100 microns eventually disappeared after 60 minutes and can be recycled and chemically degraded.

### 5.4. Other Applications

**Figure 15:**
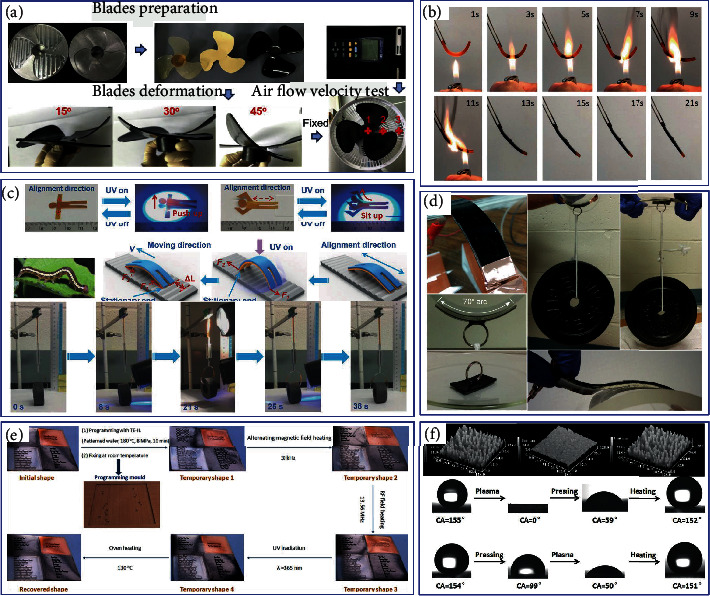
(a) SMEPC deformation and wind speed testing process. Reproduced from Ref. [147]. (b) Prototype design of SMEP fire damper triggered by flame. Reproduced from Ref. [190]. (c) SMEP double-layer membrane adjustable lightweight LCP crane. Reproduced from Ref. [103]. (d) Conductive SMEP composite adhesive. Reproduced from Ref. [191]. (e) SMEP as an intelligent information carrier. Reproduced from Ref. [192]. (f) Superhydrophobic microstructure of SMEP surface. Reproduced from Ref. [194].

Due to the excellent mechanical properties of fiber-reinforced shape memory epoxy composites, the ability to delay crack propagation can be delayed and the modulus of the system can be greatly improved. For example, the blades of fan motors can automatically switch shapes according to environmental changes, and the blade shape will also bring different kinetic energy conversion and efficiency [147], as shown in Figure 15(a). Feng et al. [190] developed a recyclable flame-triggered SMP using the flame retardancy of SMEP. This material can replace heat detectors in fire alarm systems and be used in light engineering structures with many potential fire hazards, as shown in Figure 15(b). Lu et al. [103] made a double-layer film of the carboxylic acid epoxy system into a light-operated LCP crane with a high lifting capacity. After turning off NIR and UV light in turn, the telescopic arm returns to its extended state, releasing the subject. Jeffrey et al. [191] used CB as a dopant to form a composite adhesive based on a SMP based on conductive epoxy, as shown in Figure 15(d). Applying a weight to the center of the strip creates additional local stress concentration at the interface, which reduces the apparent adhesion. Li et al. [192] constructed four physically compatible function blocks based on SMEP through physical compounding of different response materials, as shown in Figure 15(e). Different codes can be lithographically programmed to hide the initial code in response to additional information under different stimuli, providing a mechanism for intelligent information carriers. Pretsch et al. [193] ablated and dyed the surface of thermosetting and thermoplastic resins with shape memory, respectively. By engraving QR code information on the surface-colored SMP through “guest diffusion,” two different shape programming routes can be easily applied to decode the QR code, yielding an excellent information carrier in SMP. Lv et al. [194] for the first time formed a superhydrophobic microstructure on the surface of an SMEP substrate. The microstructure of the surface was broken and damaged and became superhydrophilic, as shown in Figure 15(f). After heating, it restored itself to the superhydrophobic surface. It can therefore be used in many applications, such as self-cleaning coatings, microfluidic devices, and biological detection.

## 6. Summary and Outlook

**Figure 16:**
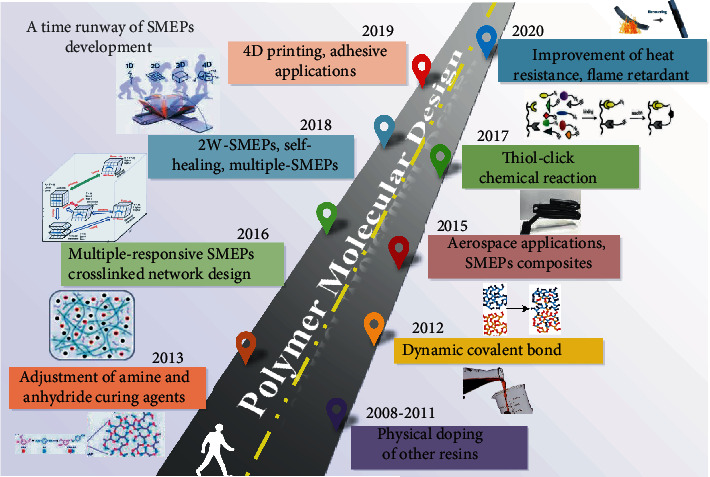
The time runway of SMEP development.

In this article, we reviewed various SMEP formulation systems and found that the functional realization of SMEP depends on the EP internal crosslinking, external structural design, and doping of functional materials. Various properties of the composite material can be improved using thermoplastic components, inorganic substances, carbon fibers, and carbon nanotubes. SMEP is widely applied in aerospace, intelligent information, 3D printing, and biomedical systems, as shown in Figure 16.

1. The performance of the epoxy resin depends on the type of epoxy resin, the curing agent, and the curing process used. The shortcomings of epoxy brittleness limit its selectivity and durability in applications, so it is meaningful to study the transition from rigid to flexible internal crosslinking of EP. Due to increasingly serious environmental problems, the development of green materials is a development trend. Biobased epoxy (e.g., rosin-based epoxy resin and gallic acid-based EP.) with shape memory also have degradable and recyclable properties
2. At present, the reversible recovery characteristics of two-way shape memory polymers (2W-SMPs) have great potential in biomedical, driving sensing and other fields. In recent years, bidirectional SMPs have been synthesized with the development of the SMPs mechanism. However, the research on 2W-SMEPs still has more potential. At present, the need for external forces limits the application space, so it is actual bidirectional shape memory performance that different shapes are given by stretch strain and *T*_trans_
3. With the development of manned space and deep space detection technologies and the construction of space stations, the intelligent design idea of material-performance-function integration is becoming more and more important. By overcoming the problems of traditional materials, the study of SMEP's multifunctional and high-performance integrated composite materials is critical in the aerospace field. Taking advantage of multifunctional combinations of materials to achieve synergistic enhancement of material properties, it also provides opportunities for new functional materials

## Figures and Tables

**Table 1:** Types and characteristics of shape memory polymers.

SMP	Research team	*T*_trans_	*R*_*f*_	*R*_*r*_	*ε*_max_
Epoxy resin	Leng et al. [121, 122]	37-162°C	≥98%	≥97%	2.2% (*T*_*r*_)
	Xie et al. [51]	40-100 °C	≥99%	≥97%	202% (*T*_*h*_)
	Biju et al. [66]	30-82°C	≥95%	≥94%	——
	Williams et al. [123]	37-41°C	≥98%	≥95%	90% (*T*_*h*_)
	Liu et al. [57]	110-180°C	≥98%	≥98%	3% (*T*_*r*_)

Styrene	Leng et al. [124]	50-90°C	≥95%	≥95%	204% (*T*_*h*_)
	Mosiewicki et al. [125]	22-91°C	≥84%	≥94%	32.8% (*T*_*h*_)
	Larock et al. [126]	30-109°C	≥97%	100%	160% (*T*_*h*_)

Cyanate ester	Leng et al. [4, 127]	156-259°C	≥97%	≥95%	8.9% (*T*_*r*_)
	Biju et al. [128]	55-157°C	≥85%	≥85%	——
	Zhao et al. [129]	91-164°C	≥99%	≥99%	5.8% (*T*_*r*_)
	Li et al. [130]	150-250°C	≥98%	≥98%	8.9% (*T*_*r*_)

Polyimide	Leng et al. [5, 131]	321-323°C	≥98%	≥98%	——
	Yang et al. [132]	272-350°C	≥98%	≥98%	56% (*T*_*h*_)
	Zhao et al. [133]	250-279°C	≥99%	≥93%	155% (*T*_*h*_)

Bismaleimide	Leng et al. [17]	95-105°C	≥95%	≥95%	17.8% (*T*_*r*_)
	Gu aijuan [134]	220-300°C	≥94%	≥88%	14% (*T*_*h*_)
	McClung et al. [135]	110-144°C	≥85%	≥99%	——

*T*_*h*_: high temperature or transition temperature; *T*_*r*_: room temperature.
